# Holding Up a Democratic Facade: How ‘New Work Organizations’ Avoid Resistance and Litigation When Dismissing Their Managers

**DOI:** 10.3389/fpsyg.2022.789404

**Published:** 2022-05-06

**Authors:** Johanna L. Degen, Massih Zekavat

**Affiliations:** ^1^Department of Psychology, European University of Flensburg, Flensburg, Germany; ^2^Department of Work and Organizational Psychology, European University of Flensburg, Flensburg, Germany

**Keywords:** dismissal, constructive dismissal, participatory observation, new work, ethnographic research, democratic organizations, organizational ethics, introspection

## Abstract

New work is used as a general term to summarize professional developments in contemporary work style, structure and modus of organizations and society—this means collaborative work and flexible working hours on individual levels, and flat hierarchies and participatory decision-making on organizational levels. Contemporary corporations strive to orient toward the concept of new work to keep up with stakeholder demands, for instance in their branding strategies as an employer. However, studies on organizational practices indicate that alongside explicit values and agendas, organizations tend to slyly exert power to secure their (economic) interests. Constructive dismissal is one such instance where contractually protected employees are made to resign their positions because the work environment is altered to become increasingly unbearable. This research analyzes two case studies to explicate routine dismissal procedures at the managerial level in two internationally operating German corporations. Both corporations explicitly profile as new work environments and are structured according to democratic principles including flat hierarchies, feature institutionalized diversity management including control committees for equal opportunities, and emphasize values such as workplace dignity, employee agency, and equality. The data contain long-term participatory observation collected over a 6-month period from two managers of 5 and 8 years of experience in managerial duties. The content analysis of data reveals characteristics of everyday processes in these organizations especially in terminating managers. The findings are presented as the ‘*model of the silent dismissal*,’ containing seven types of managerial termination carried out by implicit power and symbolic conventions that circumvent subject participation and litigation in an effortless manner. After exposing the model’s mechanisms, we turn to discuss its meaning for both terminated and surviving subjects against a critical theoretical framework of neoliberalism, democracy, and power.

## Contemporary Work and Dismissals

### Democratic Values Under a New Label: New Work in a Contemporary Corporate Context

Various contemporary developments in labor relations across the western neoliberal world are changing due to globalization, digitalization, and the way of work calling for flexibility, autonomy, collaborative and entrepreneurial working style; these changes are described under the rubric of new work (Aroles et al., 2019). The change toward new ways of working and the label of new work symbolizes a promising, progressive future and a strive toward new (humanist) values within the economy at multiple levels. It is the ideal, founded in the 80s by Bergmann (1996, 1999), of true freedom of the individual that really does what one wants, where new work means the end of burn-out, exhaustion, and alienation (Behrend and Brohm-Brady, 2020). New work collects optimistic and visionary characteristics for *the* future of work, including learning, meaning, and shared knowledge (Schnell and Schnell, 2021), a work environment that pays explicit attention to individual needs and agency, freedom, intrinsic motivation, prioritizes flexibility in time and space, participatory processes and ethical subtleties (Brommer et al., 2019). New work is thought of as a revolution of work leading to safety, health and satisfaction (Handel and Levine, 2004). On an individual level, therefore, work becomes a place of identification, self-actualization, and existential security in a neoliberal social context in which liberated subjects are responsible for their personal success, happiness, health, and meaning-making (Rose, 2006). Subsequently, a subject’s perspective on work shifts to an urge for identification and implied meaning with their profession and workplace, including new demands and expectations. Therefore, the impact of new work extends from the individual to the organizational levels (Cappelli and Keller, 2013; Petriglieri et al., 2018). On the organizational level, this means restructuring and reorganizing work in terms of flexibility regarding schedules, clocking-in and place as well as a drive for innovation in terms of the transformation of hierarchies to integral organizations characterized by collaborative and entrepreneurial working styles (Hackl et al., 2017; Aroles et al., 2019). Corporations usually draw on long-established traditions on how to operate and thus contemporary corporations feel threatened by new work demands and fear losing control (Aroles et al., 2019). Nevertheless, omnipresent confrontation with ever-changing conditions and demands in a competitive market, as driven for instance by stakeholder demands (like in branding as an employer), compel corporations toward the new work principles of work. These principles include flat hierarchies, participatory decision making, equality in the workplace, autonomous leadership approaches and fairness (Hackl et al., 2017; Aroles et al., 2019). These developments point to the future of work and its values and apparent tensions between subjective and organizational levels. Moreover, the transition from traditions and established practices to new values and principles underline the significance of studying the actual implementation of such claimed values alongside everyday processes. These lead to questions such as: What do new values mean for subjects and how are new values carried through and implemented in routine organizational practices? How far are organizations genuinely engaged with the values of new work, or merely resorting it to secure their interests?^1^

Critical research on new work has so far focused mostly on effects at the subjective level. Besides the seemingly positive effects of freedom and autonomy, these studies show ethically questionable aspects from the standpoint of the subject like the loss of privilege and assurance, extensive work engagement and eroding borders between professional and private spheres (Marvakis, 2019). So far, questions about practical and concrete transposition in everyday-, (micro-) processes on an organizational level and effective aspects of power and control have gone largely unnoticed in mainstream approaches to the study of the transition to new work (Aroles et al., 2019).

### Organizational Processes: The Example of Dismissals

Managerial and CEO dismissals are well researched; however, previous studies concentrate predominantly on investigating explicit processes, conditions, and measurable effects. These studies measure for instance the subject’s performance and the investigation of who is typically dismissed (Park et al., 2014), justifications, reasoning, and the respective impact of terminations on work environment (Harcourt et al., 2013; Strandholm et al., 2013; Kim, 2014; Li et al., 2017; Wang and Yang, 2019), structural conditions such as terms and conditions of contracts, and external factors as the role of stakeholder pressure in dismissals (Brockner et al., 1986; Boeker, 1992; Largay and Zhang, 2008; Wiersema and Zhang, 2011; Park et al., 2014).

The legal model of employee dismissals can be challenging for employers in Germany as *hire and fire* policies in the country are protected by highly formal and legal standards (de Vaate, 2017). Consequently, implicit forms of dismissals tend to emerge in order to secure organizational interests within the strict legal framework. One such example is the general and increasing tendency of employers to engage contingent work, rather than permanent work contracts or subcontracts where dismissal *per se* is circumvented. These strategies however mostly, but not solely, affect early-career employees and lower positions in organizational hierarchies (Voßemer, 2019).

In addition to the legal intricacies, terminations also involve ethically questionable practices such as constructive dismissals. Constructive dismissal is a rather equivocal practice, whereby employees are driven to resign because their work environment is made increasingly unbearable (Dickens et al., 1984; McCall, 2003; Davidov and Eshet, 2015; Chen and Chen, 2017; Popovic, 2018; Suciu, 2019). Such an environment is created through mechanisms including social exclusion, harassment, degradation, suspension from communications, a deliberate mis- or reinterpretation of contractual commitments (Westhues and Jansen, 1999; Poulston, 2005; Reynold and Palmer, 2005; Mann, 2006) and—on a higher hierarchical level—cutting a manager’s budget and reducing their staff (Westhues and Jansen, 1999; Poulston, 2005; Mann, 2006). Although constructive dismissals formally can have legal and financial consequences for employers, it remains difficult for the plaintiff to prove their case. Employees affected often do not resort to legal action as they feel powerless or are simply uninformed. Usually, they receive little help or advice and the potential compensation is often relatively small, while the chances of being socially stigmatized are perceived to be high (Voll, 2007). Constructive dismissals are therefore rarely reported; and even when it is extremely difficult to arrive at a verdict because the court can hardly determine whether the employee has willingly resigned or has been slyly dismissed (McDonald et al., 2008; Dekker, 2012). Most studies on constructive dismissals focus on vulnerable groups such as pregnant women (Voll, 2007) and employees and managers at lower hierarchical levels as being affected by constructive dismissal.

### Research Question: Processes in Democratic Organizations, the Case of Managerial Dismissals

This research argues that the way new work impacts and regulates power structures and relationships are not always libertarian and transparent despite its democratic origins and pretensions. Alongside the processes of managerial dismissal, this study explores everyday practices in contemporary corporations. The analysis reveals the complex role of power and control in supposedly democratic—new work—organizations and seeks to answer the question *How do large corporations co-opt routine democratic organizational procedures in dismissing (visible) managers in order to evade the consequences of making direct decisions and taking explicit actions?*

In what follows, we explain the data collection as participatory observation, concrete procedures, and methodology. Then, we will explicate the findings of ‘silent’ managerial dismissals. Finally, the model of silent dismissal and its seven types of managerial dismissals will be proposed. The conclusion will locate the model in the ongoing discussions about democracy and its relationship with power and economic interests.

## Method: Exploring New Knowledge Through Everyday Practices

### Data Collection: Organizational Participant Observation

Degen (2022) found indications of constructive dismissals at managerial levels when conducting 75 interviews in 2019 and 2020 to research everyday practices of diversity management. In these interviews, constructive dismissals were an incidental yet recurring phenomenon. The participants repetitively describe subtle dismissals carried out by implicit yet effective mechanisms in processes of termination as prevalent common practice: “*to dismiss somebody we [i.e., the head management] do not have to do anything, it’s the teams and the dynamics that make them leave, easy*” (case 3), “*the real question is not who is dismissed but who is leaving, that is sometimes very blurry, organizations have their ways*” (case 7), “*[in the context of dismissing employees] There are informal processes in place that are hard to explain, but they are very effective*” (case 5).^2^ Nevertheless, as the constructive dismissal was not the focus of the former study, insights remained relatively general and in the margins of the analysis. This observation, however, intrigued the authors to further explore the occurring phenomenon in detail. Two managers from the sample of the previous study who mentioned dismissals agreed to participate in an advanced in-depth data collection in 2020 to explore silent dismissal and its characteristics.

These two managers share some similarities in terms of being on a corporate career track in internationally operating corporations in Germany (revenues at about 17 and 600 million EUR), yet they are operating in different sectors. The careers of both subjects had started with an internship after a master’s degree in management studies. Being promoted through several hierarchical levels and departments, they eventually landed upper managerial positions just below executive management. Manager A has 8 years of experience and leads a team in the department of online-marketing, while manager B has 4 years of experience and serves in a leading position in the HR department. Both corporations are long-established economic players, actively addressing and implementing politics and principles as new work environments, explicitly named (on their online representation) including flat hierarchies, participatory decision making, fairness, equality, and democratic processes. Both corporations are members of the diversity charter ‘Charta der Vielfalt,’ an organization that explicitly promotes equality in the workplace. To preserve the anonymity of the managers, we have decided to withhold further details of their careers and the corporations.

To explore silent dismissal, we designed a data collection procedure in the form of participatory observations including autoethnographic journals and complementary expert interviews. Autoethnography is an appropriate method for the collection of data in implicit and possibly ethically sensitive practices, because it allows access to sensitive topics from a first-person perspectives that can “(…) *provide first-hand accounts of taboo topics such as sexual harassment and bullying and (…) various moral dilemmas and highly charged emotional situations in the workplace*” (Boyle and Parry, 2007, p. 189) and thus can shed light on experiences that usually remain concealed (Ellis and Bochner, 1996, p. 25). The first-person perspective to access knowledge follows the tradition of critical (work) psychology and the standpoint of the subject^3^ and their well-being (Schraube and Osterkamp, 2013; Bal et al., 2019; Weber et al., 2020). The critical paradigm and approach of data collected enable insights into a complex, real-life context as the data are neither limited to academic language or scope nor deductive questions and presumptions (Gläser and Laudel, 2013; Schraube and Osterkamp, 2013).

Prior to collecting data, the authors of this paper had a conversation with the managers to provide a guiding impetus and overall orientation including a method of introspection building mainly on Vermersch (2017) evoking internal witness and techniques of Albrecht (2020) to observe social dynamics as an external witness. These techniques include an explicit acceptance of one’s own subjectivity and reflection on social norms and desirability, just as sensitive wakefulness when observing social processes, alongside the following guide:

1. Accept to trust in and share your subjectivity, experiences and observations to the fullest.
2. Start by focusing on concrete situations.
3. Remember the lived experience by mobilizing the evocation act, a necessary condition to access the detail of the past lived experience.
4. Verbalize the lived experience descriptively, avoid commenting and contain the verbalization of inner processes, context, or circumstances.
5. Accept and explore fragmentation of the action’s description to gain access to the level of detail which will generate the elucidation of the development of the action.
6. Aim for the amplification of the qualifications to go further than the summary judgments, like “it was good,” “it was hard,” “it was nice.” Include your perspective on how and why.
7. Try to challenge what would normatively and habitually be pleasant, impressive, or correct to express.
8. Pay careful attention to the completion to know if all the information necessary to fully understand the action, from the beginning to the end.
9. If you feel like it, add other situations, meta thoughts, or personal conclusions after the emphasized situation.

Data collection ran over 6 months, when the two managers (A and B) continuously took note of their observations both in their current position and previous employment. The impetus invited them to ‘closely describe personal observations of various processes such as hiring, decision making in general and promotions and with a main focus on (managerial) dismissals, including all details available regarding actual changes, indifferent changes and subjective perceptions of the managers themselves and other actors’. They were asked to include all observations using all senses alongside the guide above and maintain the journal at least two times a week but as much as you feel like it. There were no formal prospects in terms of style or extent. The journals were offered as blank sheets in a booklet but could also be conducted digitally using a text program, and both formats were used. Manager A used handwritten notes and digital text applications, manager B used digital formats and emails to capture observations, often like field notes, which then were further elaborated on in dialogue in the interviews.

During the six months, we carried out three interviews with each manager, respectively, after the months two, four and six (case A interview 1, 2, 3, and case B interview 1, 2, 3) in which we scrutinized the observations alongside the notes from their journals. The interviews were conducted as explorative expert interviews (Gläser and Laudel, 2013), where the interview unfolds as a coequal communicative dialogue between the interviewee and interviewer with little prefabricated (deductive) guidance. The interviews ranged between 64 and 98 min in length and served to explore the subjectivity of the participant and put a distance between the authors interpretations and presumptions and the participants’ point of view. The reflection collected in journals did not suffice because they tended to be rather general notes and open to interpretation. The interviews, therefore, serve to clarify and deepen the journal notes, which again function as anchors and remembrance.

Journal note by manager B: “Colleague disappeared without notice and it felt cruel and shocking, saw maintenance remove name tag, no further communication.”

Interviewer read out loud the note, then asked: “What did this mean for you?”

Manager B: “I felt empty, sad, and afraid because this could have been me and my whole dedication and years of loyalty gone down the drain and also I was wondering about where he is, so this is a source of concern and anxiety for myself and empathy for the other.”

Interviewer: “What exactly do you mean with ‘no further communication?’”

Manager B: “I mean that there was no official statement, no email and no meeting, no one really mentioned this happening, it was very odd, we only had one private quick chat between a close colleague and me, that’s what I mean.”

Journal note by manager A: “Everyone acts weird ever since the incident, the heavy atmosphere, one could cut through it, everyone acts happy though, this makes me feel weird and crazy. Asked Astrid, she feels the same, makes me feel sane again.”

The interviewer read out loud the note then asked: “Which incident do you refer to here, and what time frame are we talking about?”

Manager A: “The disappearance of a manager room on the floor below, the atmosphere had changed for weeks, this was around two weeks after the disappearance, everyone acts just unusual, conscious and very formalized, if this makes sense. We all tried to make no mistake, say and do nothing wrong and that resulted in this weird atmosphere, where everyone acts slightly off, just like after a fight with a partner, where the wounds are not healed yet and the problem is not really solved.”

Interviewer: “I see.”

### Gaining Inductive Insights by Using Mayrings’ Qualitative Content Analysis

The analysis of interview data was carried out by using qualitative content analysis following Mayring (1991, 2015). Qualitative content analysis usually follows two principles: deductive creation of categories based on theoretical assumptions and theoretical knowledge-based questions and inductive category building where the categories originate strictly in the data (Mayring, 2000). Following an explorative approach, we focused on the inductive categories. The process followed four circulating steps of (a) finding overall occurring themes and naming them descriptively as topics, (b) organizing occurring codes including the data sequences, (c) summarizing those codes to more abstract categories and subcategories, and (d) finally building a codetree with categories, differentiating subcategories and exemplary codes. The building of the codetree is followed by going back to coding the comprehensive data material based on the codetree. First, two researchers individually studied the data to determine topics chronologically. Then, all topics and aspects were (re-)arranged, identifying abstract categories and respective subcategories including codes and precisions in order to develop an unequivocal codetree (Mayring, 2000). Subsequently, the researchers met to compare and synthesize their analyses. A research assistant used the final codetree (see the comprehensive codetree in the Supplementary Material) to go through the whole dataset to code and sort the comprehensive data in several loops.

## Findings: The Silent Dismissal

As findings we first describe the ‘silent’ dismissal including characteristics and mechanisms of expelling managers (Section ‘How to Dismiss Managers Silently’). Second, we depict the typology of dismissals that emerges from the results of the analysis and describe the types of dismissals as a model (Section ‘The Model of Silent Dismissal’).

### How to Dismiss Managers Silently

The process of managerial dismissal is explained and interpreted along the main categories, respective subcategories and typical instances of the process including indicators, mechanisms, effects and behavior within the context of the characteristically silent dismissal. The findings are elaborated including extensive data examples and subcategories, following the six main categories, including (1) formal indicators and process of dismissal, (2) circumstances, (3) communication, (4) (power) mechanisms, (5) initiation and direction, and (6) (re-)active behavior.

1. Formal indicators and processes of dismissal: ‘*Ghosting without warning or even a formal promotion*’

Indicators of a silent dismissal can be visible or invisible and differ in regard to the target group. An example of a visible and progressive indicator can be the assignment of new office space. This space is often of a lesser symbolic status, for instance from a corner office or a top floor to a lower floor, smaller office, and most often not in the proximity of top-tier managers and leading management. This change comes in varying expulsion. In some cases, managers are even relocated into a supplemental office space, such as an open-plan office usually used for freelancers.

“*I observed how this manager on the executive level, I mean he once hired me, simply was banned. He was relocated from his prestigious office and had no place of his own anymore, he was sitting in the office-plan. He had to find a desk every morning among intermediate staff and interns. I do not know if there is anything more embarrassing. Before, he had a corner office on the top floor, I mean what a cliché and from one day to another, he was sitting there and with that he was kind of erased in general. I tried to talk to him, but he was taciturn, he tried to become generally invisible, maybe due to the embarrassment. I would not know how to react, it was very awkward for all of us, everybody actually avoided him ever since*.” (A 1; 239f^4^)

The change of office space often goes hand in hand with a change of job title^5^ or a change of the formal position. Strikingly, the new title or position is either objectively on the same level or formally a promotion and is associated with seemingly attractive offers like promising new projects, impressive titles or position descriptions. The demotion is not readily visible; it is hard to grasp and it seemingly takes several years of experience to learn about such mechanisms, as they resemble actual promotions and career developments.

“*It is most often a promotion, or something new, seemingly exciting and simply better looking. There is this info and in the beginning I was thinking okay, this sounds quite awesome. A new project or a new job description or a title. I learned over the years that it is a promotion but sometimes I never hear of those promoted again. I learned to sense if it is a real promotion or a dismissal, but I’m not yet always sure. (…) No, actually that is not true, because we can definitely feel it. Maybe it is because true promotions are longer processes, everybody follows along and it’s known. If it is sudden and unforeseen, it often is a handshake out [meaning: to leave the corporation]*.” (B 2; 516f)

These visible changes are accompanied by invisible, implicit intersubjective changes, for instance social exclusion. The social exclusion can be either in the case of official events or informally arranged occasions.

“*From one day to the other we just, I do not know why, did not pick him up for lunch. When he saw us eating, he would sit by himself not approaching us, it was actually odd, before we have been professionally close*” (1, 190f).

Other forms of social exclusion include the manager being cut off from communications such as emailing lists or their access being restricted to specific spaces and groups on the intranet. Besides the communication, there are indifferent and vague changes described as a changing ‘atmosphere’ steering everyday behaviors and even personality on a micro-level.

“*There was one manager, he was always noisy, present, we all knew, noticed and respected her. She had quite an aura, she was always having an opinion, she was pushy, often helpful(…). She dared to critique and her team was always a step forward. When the process started she turned quieter from day to day, it was like a change of everything she was before, her stamina was gone, her self-confidence gone. We all knew what was happening and so did she. She was silent for several weeks, then she disappeared the usual way*.” (A 2; 240f).

Visible indicators of a manager being dismissed can alternatively be sudden. In contrast to the progressive ways described above, these are abrupt; managers in this case would vanish from one day to the next. All their personal belongings, name tags, and profiles and contact information on the website are erased, often overnight.

“*I came to work and saw the agency removed the furniture and personal stuff from my colleague’s office, we never saw him again and we never talked about it. He was really good, performing better than me. This left me anxious.*” (B 2; 601f)

Other indicators are hidden in the institutionalized restructuring processes. Organizations are even willing to modify their structure regularly in order to make a manager and their position redundant. Here, the position of the manager becomes obsolete and the manager disappears in the process.

“*The restructuring is the dice game returning biannually. It means the whole corporation restructures departments. It’s usual, still stressful. Often the putative reason is to become more effective, to save money, to become a digital company, there can be many reasons. What it really is about is keeping the employees busy. There will be new positions and task descriptions and then we all have to apply for our own positions. And through this, the corporation has the opportunity to let people go.*” (A 1; 615f)

2. Actual circumstances and key points: ‘*A dream situation with no tasks and full salary*’

The second category of describing silent dismissals concerns measurable work conditions such as salary, staff, tasks and budget. These fall into three broad categories: (1) the conditions that change by a decrease in resources and/or duties; (2) the conditions that change by an increase in resources and/or duties; (3) those that remain the same.

The managers continue to receive a decent or even high salary. They often remain their technical features such as devices. At the same time, the requirement of physically showing up at the office is upheld. However, removal or reduction of certain key points such as staff, responsibility, budget, objective and concrete tasks, attention, procuration, visibility, and their voice is excised. They no longer attend meetings or similar events. This means managers have ‘empty’ projects, without a budget, with no one to report to, and with no staff. Those types of ‘empty’ projects can be a strategy paper or online tools, which will never be implemented. These tasks serve as a sort of façade. This could be perceived as a dream situation, free time, no responsibilities and a full salary. Nevertheless, the situation of being (professionally and personally) ignored and unacknowledged by symbolic (and masculine) means seems to be perceived as a heavy burden making managers resign after a short while.

“*These degraded individuals have to be present but what this person does, does not matter, they quit quickly, nobody stoops to that. Except, I know ONE (loud) guy, he would not leave, he had his salary and was sitting in the open plan office for FIVE (loud) years. Nobody knew what he was doing and nobody asked any questions. Later I learned he established a company on his own. He was the only one who just hung in and did not quit. Today I often think about him, he developed his own agenda and did not let the situation within the company get to him. Actually, a dream, sitting there on a management salary and working on your own stuff, but I never observed anybody else who could ahead of the situation and managed to turn it into something positive. It is still cheaper for the corporation to pay the staff and wait it out, especially because of the public pressure, avoiding public drama is the primary aim.*” (A 3; 735f)

On the organizational side, the readiness to face economic expenses indicates the value of the silent, implicit procedures, indicating significant advantages in terms of organizational interest.

“*This is the perfect strategy, you cannot file a complaint or have a lawsuit about being promoted (laughs) or getting a raise, harassment you can report, this: impossible.*” (A 3; 791)

3. (Non-)Communication or praising phrases: ‘*Wishing them all the best*’

These dismissals are accompanied by specific methods of communication. While silence is most common, there are explicit forms of communication, too. Often there is a tacit agreement or understanding of not mentioning anything and asking no questions. This means that both the dismissed, the survivors, and management do not speak (up) during the process and avoid referring to the incident. And there is no official statement about the dismissal.

“*One day I showed up and met the janitors who were removing my colleague’s name from the office door. I mean, all was still good the previous Monday. That was the day it got to me, how disposable we all are. We deliver everyday all day and dedicate our lives, but from one day to another you might be gone and some janitor scrapes your name off a door and what was once your achievement of many years would just be another white door. And we did not ask questions and we never mentioned it. I mean, I just told one colleague I know privately how shocked I was, when I met her after work at the gym, but it was more like ‘did you see that’ and she said ‘yes, I never saw that coming’. That was that conversation*” (A 2; 314f)

The second method of communicating a dismissal is characterized by general phrases typically containing no concrete and specific information. This form of communication is usually described as empty phrases and general clauses.

“*Sometimes there are emails formally informing about supposed promotions or when somebody leaves. These emails always sound the same: ‘We thank xy for the effort he put into our company, we are very grateful and wish him all the best. Please welcome yx and add him to the emailing list.’ Similar phrasing occurs in the form of press releases.*” (B 2; 580f)

4. Power mechanisms: ‘*The Sword of Damocles*’

Two parties are usually affected by silent dismissal. First, the managers who are made to resign their positions partly due to stigmatization; second, the colleagues of the dismissed manager as remaining survivors. The data show that the dismissed seem pushed out by symbolic mechanisms such as lack of attention and acknowledgment, uncertainty about the reasons of being stigmatized, social and informational exclusion, overall degradation and perceived stigmatization, and the burden to find meaning in self-constructed work objectives.

“*Actually, as I said, you can do nothing and will be paid, but still people suffer and leave. I would say it never takes longer than two months maybe four. I mean these people are used to being cherished, respected, required and looked up to, they, or we whatever, feed on this power. Sitting there in public disgraced, embarrassed, with nobody talking to you does something to people, and they quit and that of course is extremely advantageous, cheap and easy for the corporation.*” (B 3; 812f)

Observing these everyday practices has a strong effect on the remaining employees. They reconsider if they are still in favor and how they can remain so. There is a general fear of being disfavored and losing one’s existential basis and position, despite the fact that the actual agent is often unknown or disguised. This omnipresent insecurity and favor/disgrace dichotomy of status are described as “Sword of Damocles” (B 3; 81). Precarity is the drive that fosters subject compliance. Besides the fear, it seems detrimental to observe colleagues to be dismissed by implicit power mechanisms and, therefore, being in incongruence with the values of the corporations.

“*It got to us. In the beginning I thought excellent performance would secure my success. It takes a while to learn because it is subtle. My career was going very well and I was close to the executive management, but I always wondered if or when it would hit me. I actually trusted very few people. How would you explain that nobody stands up, that is something I still wonder about? I know nobody would stand up for me either, that’s where we spend our lives. But I admit, I never did either, not before today with this project. Still I feel like a whistle blower. It feels WAY (loud) safer to stay quiet. Speaking up is direct career suicide. So I keep working and hating what’s going on.*” (B 3; 689f)

5. Initiation and direction: ‘*The atmosphere just changed*’

While usually dismissal follows a top-down logic, such as being dismissed by the board, the initiator remains mystified in silent dismissals. This can be for instance by a general restructuring process supposedly aiming for organizational development or general blurriness, where there is seemingly no identifiable initiator or decision maker. It might seem that reason behind this obscurity is that the participants did not have access to final information; however, their management level was only one level below the board. It seems rather unlikely that the corporation’s board is explicitly deciding about these dismissals. Instead, it is indicated that the dismissals are implicit, collectively supported dynamics with no clear or identifiable single initiator. The initiation instead is perceived as some implicit change of atmosphere, where compliance is part of the mechanism.

“*Sometimes it seems like micro actions causing the change. I know one intern who sent an email, and emails in our corporation have to be polite with a specific elegant tone, often we write them together as a group or even with the whole department. All my interns have to get their emails cleared by me, this is highly political. However, I heard that one person sent an email missing to address the status of the person correctly, and that person was gone shortly after. This can also be because she was simply not very good and the email was a symptom, but it is an example. It was not her team leader dismissing her, it was like, there was simply no future for her. And that is characteristic of the general process. Often it is neither the direct supervisor nor the head of the department. It is not clear where it comes from, it is like this butterfly flap and then suddenly the tide turns. I doubt the board meets and turns the thumb up or down about every single annoying team leader (laughs)*” (B 1; 1030f)

6. Re-active behavior: ‘*We remain quiet and unobtrusive*’

The reactions of all subjects, dismissed subjects and remaining employees, constitute a part of this dynamic. Both groups accept the process and comply. There are a few exceptions that are reported. Besides condemning the dynamics from the side of survivors who insist “*we all hated it, I feel one has to talk about this*” (A 3; 260), there is the case of one subject building his own company on his managerial salary (see above) and one manager working her way back up. These exceptions were constituted by the subjects’ restricted agencies, refusing to succumb to the role of the disgraced.

“*There was one exception, it was a woman who had a solid network. She chose to remain in the company although she was demoted. Today she is the head of a department. It took her five years, but she somehow remained in the corporation. She changed the department several times and was very engaged, holding her head up high and she came back. I noticed that she was always extremely well dressed and held it together in terms of posture and appearance. She was powerful before, usually that exaggerates the stigmatization but, in this case, she could mobilize her former contacts, maybe because she never gave up.”* (A 1; 989f)

### The Model of Silent Dismissal

**Figure 1:**
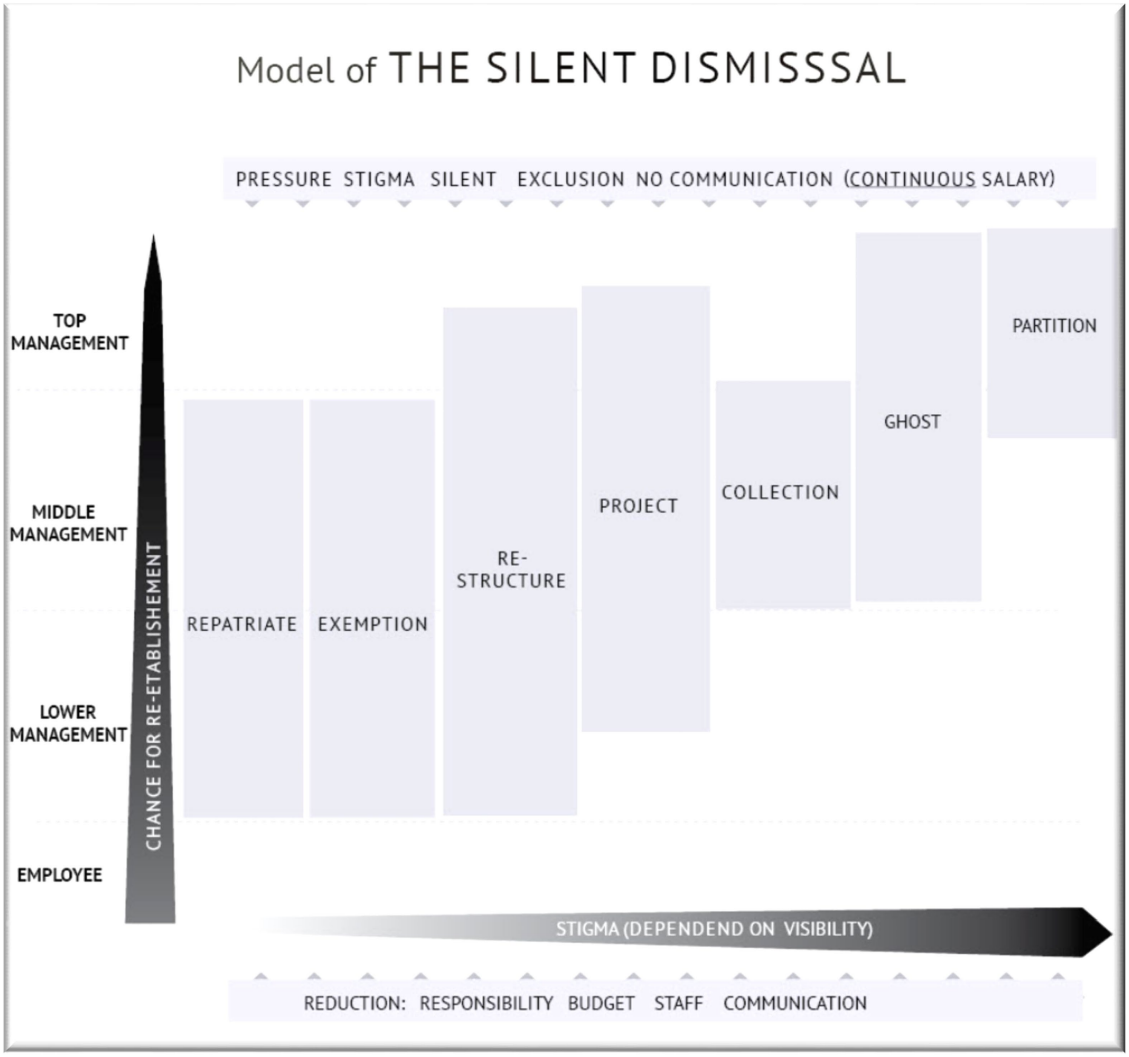
The model of silent dismissal.

These general methods of silent dismissals lead to a typology containing seven types. The types are differentiated along the indications and mechanisms during the process and the target group (Figure 1).

#### Type 1 Repatriation Dismissal

The repatriation dismissal concerns managers who find they are redundant after returning from a position abroad. As the stigmatization is relatively low, they are able to stay within the company and reorient, leave the company to obtain another position in the same sector, or leave again for yet another position abroad. This type of dismissal applies mostly to lower and middle management. The level of stigmatization is the lowest, as the cause is most often attributed to the situation of being absent, rather than personal qualifications, competence or responsibility. Managers subject to this type of dismissal are usually disregarded and their needs are not provided for. There is no communication about the process.

#### Type 2 Exemption Dismissal

Lower and middle management are usually subject to the type of exemption dismissal that features relocation or exemption and is characterized by being relocated and exempt from tasks, denied office space, staff, responsibility, and budget. This is demonstrated by the open plan office example, where a former manager has to work among freelancers and interns. This dismissal is induced mainly by the removal of objectives, isolation, and relocation. The level of stigmatization is significantly higher as the managers are visible and are observed in the degrading working space on a daily basis by colleagues and former staff. There occur forms of non-communication and communication as a promotion or assignment of a new project.

#### Type 3 Restructuring Dismissal

The type of restructuring dismissal is implemented by strategic and repeated general reorganization, where managers have to reapply for their own or new positions and these applications can be rejected on the basis of recently changed circumstances. All management levels with the exception of the CEO are subject to this type of dismissal. The level of stigmatization is high as the subject’s work objective and skills are becoming superfluous for future prospects. Yet, there is a chance of being reestablished in other departments and the same industry. Nevertheless, the more visible the manager used to be, the lower their chances of reestablishment. There is no accompanying communication.

#### Type 4 Project Dismissal

The project dismissal is disguised as assigning a new project, position, a new title and sometimes a seeming promotion. The new position, however, is ‘empty.’ The manager does not have access to budgetary resources or staff and can be relocated into a less desirable work space. This type of dismissal applies to middle and higher management and is highly stigmatizing. Reestablishment is unlikely. This type of dismissal is communicated explicitly as a new position or promotion.

#### Type 5 Collection Dismissal

The type of collection dismissal was employed in only one of the two corporations. Managers who have lost their positions or projects are pooled on a waiting list at the HR department. While waiting, they have to apply for any vacant position that might pop up and perform various tasks for which they are overqualified. As the wait continues indefinitely, they usually decide to eventually leave the company. The level of stigmatization is high, as these subjects are listed under a department declared as ‘waiting pool.’ Though low, there is still a small chance for re-establishment through finding a new adequate position. Usually, there is no communication about the process.

#### Type 6 Ghost Dismissal

Ghost dismissals are usually reserved for the upper management and CEOs. The manager suddenly vanishes. Colleagues will never see or hear of the dismissed manager again. A reestablishment is impossible and this applies to both the headquarters and industry. Often there is no communication about the dismissal or only ‘empty’ phrase communication.

#### Type 7 Partition Dismissal

Partition dismissal is used when a top-level manager has to share his position with a co-manager. This strategy is adopted when it is not possible to directly dismiss the manager as in the case of a board member or when such a dismissal can accrue a large cost. The process is often introduced by explicit public communication of a seemingly positive piece of news.

## Discussion: Neoliberal Ideology and Democratic Value System as a Context for Silent Dismissal and Its Meaning for Subjects

The silent dismissal is a ‘noiseless’ process where managers formally resign but are actually made to do so due to power mechanisms based on withdrawal of participation, change of position and access to opportunities, and most importantly lack of external acknowledgment. It functions through blemishing the symbolic status and actual power of managers, which is often perceived as stigmatizing, social exclusion and degradation. Ghosting or promoting, assigning void projects, ignoring and excluding managers in intra-organizational communications, therefore, constitute the model of silent dismissal. Formally, these dismissals come in the disguise of a promotion or hierarchical change that is legally and often seemingly legitimate and could be even called a dream situation. The salary is never withheld; in fact, often silent dismissals feature a raise. In contrast to constructive dismissals, these mechanisms are a disguise (for instance a formal promotion) coming with a friendly or neutral facade that makes it hard for the dismissed and others often to recognize and practically impossible to litigate. Nevertheless, these mechanisms require collective compliance in order to be effective.

As silent dismissal cannot be ascribed to a specific agent in the organizational structure, the mystifying dismissal process serves as a concrete threat to all survivors, hence providing a constant warning to regulate their organizational behavior. It intimidates the surviving employees into complicity and compliance. These mechanisms are implemented through different strategies—including repatriation, exemption, organizational restructuring, project assignments, collection, ghosting and partition—depending on the level and prominence of the manager and, respectively, involve different levels of stigmatization and chance for reestablishment, which is usually low. To avoid legal repercussions for dismissing a prominent manager, corporations choose to simply marginalize them and divest them of their authority. This will eventually make them resign their positions without any overt duress.

These mechanisms bear a great significance for the subject, first at the intrapersonal level in terms of degradation, turning toward the self, well-being, and health, and second, at the relational level in terms of social positioning, relations, and organizational dynamics. Social exclusion has negative consequences on well-being and integrity at the personal level and on relations, trust, identification, loyalty, and performance at social and organizational levels (Grosser et al., 2010).

Silent dismissal impacts the terminated subjects, who lose their position, experience existential threats, are cut off from social relationships, lose their sense of belonging, and experience social exclusion from former co-workers and employees. This will impact their sense of self as relational entities dependent on social relationships, validation, and overall social positioning (Robinson and Tajfel, 1997; Gergen, 2011). Consequently, social exclusion most likely affects social attitudes, such as aggression, defensive behavior, self-defeating behavior, and mistrust (Twenge and Baumeister, 2005) and health in terms of anxiety, loneliness, depression, jealousy, relaxation, self-esteem, and self-worth (Leary, 2011; Pereira et al., 2013).

The reactions that silent dismissal invokes can be understood in the context of neoliberal ideology and neoliberal democracy. Dismissed managers simply accepted the process and their colleagues complied with it without raising any objections, despite their better judgment. Neoliberal ideology is the incorporation of economic logic that is applicable both to the environment and individuals (Rose, 1992, 1996, 2006). Neoliberalism ascribes structural issues such as unequal chance, dispositions and access to justice to self-responsibility and diligence. Subjects then are held responsible not only for their career and existential security but also for their happiness and health. The neoliberal narrative perpetuates the organizing of work and its role, claiming freedom and autonomy. However, those seemingly progressive values often come with hidden disadvantages for the subjects, including precarious contracts, and insecurity (Marvakis, 2019). This means, subjects are not only responsible to constitute their own existence, producing happiness and a worthy life (to avoid being seen as a failure) but also bearing actual risks when becoming unemployed and in need of social security or support to fulfill their role of being a potent consumer (Bal and Dóci, 2018; Tommasi and Degen, 2022). Such conditions of life and work explain how the termination is interpreted as devastating and supposedly not only a threat to one’s career but exaggerated also an existential one. Instead of showing resistance, terminated subjects seem to turn against the self. Dismissed managers tend to blame themselves instead of turning to institutional structures designed to provide access to justice and equal opportunities, like leaders, staff councils, and supervisory boards to seek help and justice. Such institutions instead seem rather function as a token and are not mentioned as applicable for the subjects in need, which shows that the disarming strategy employed by corporations works effectively.

At the same time, the observant survivors are effectively threatened and react with compliance to avoid being discredited, often referring to the fear of risking their own position. What remains are speculations on how the discreditation might have been caused their situation, and projections of how devastating the consequences must be. Subsequently, the subject has to grapple with her personal values and doubt institutional justice, but still recede to inaction and complicity due to the fear of losing the own position.

This might affect loyalty, integrity, and motivation in a negative way, hence adversely impacting performance and turnover intentions. This is primarily because mistrust in organization and leadership arises from experiencing the utilization of ethical values used as a façade hiding economic interests and the business case (Tomlinson and Schwabenland, 2010, Renn et al., 2013, De Luca et al., 2017, Vassilopoulou, 2017). Both the organization and the leaders are perceived as authoritarian, not trustworthy, and a threat, acting based on hidden interests and in unethical ways. New work then, though promising a just and progressive future, subtly integrates neoliberal ideology and long-established interests and power mechanisms in order to incorporate and enforce the dynamics of the labor market, power relations, and consequently questioning the organizational claims of democracy, participation, and value-based ideas of contemporary leadership (such as participatory, transactional or transformational leadership).

Instead of seeing this as a contradiction, one might argue that the process is actually democratic because democracy aims to maintain economic interests in its historical understanding. Democracy was conceptualized as early as the fifth-century BCE, when Plato states that (Athenian) democracy is one of the major forms of government in *Statesman* and *Laws*. For him, democracy is ruled by the majority of the people. As this system of government does not rely on the expertise and knowledge of the rulers, Plato deems it inferior to other forms of government because either the rulers do not have the intellectual rigor to rule or they have to appeal to the mob to win office and pass laws—concerns later echoed by Hobbes in his *Leviathan*. In *Republic*, Plato argues that tyranny “evolves from democracy” (562a) and is grounded in economic vested interests:

*Well, is not the city [symbolizing a democratic organization] changed from an oligarchy to a democracy (…), because of its insatiable desire to attain what it has set before itself as the good, namely, the need to become as rich as possible? In what way?*

*Since those who rule in the city do so because they own a lot, I suppose they are unwilling to enact laws to prevent young people who have had no discipline from spending and wasting their wealth, so that by making loans to them, secured by the young people’s property, and then calling those loans in, they themselves become even richer and more honored.* (555b-c)

In other words, Plato believes that democracy is a form of lawlessness that secures the economic interests of the ruling class.

There is a similar correlation between democracy and economic interests in Foucauldian thought. Raffenne and Toumi (2009) argue that for Foucault, democracy is “*a network of rules, practices, and bodies of knowledge [as embodied knowledge]*” (Raffenne and Toumi, 2009, résumés). They maintain that democracy is structured so that “*rules are stretched to the best possible advantage and the deceptive tightly-knit texture of prescriptions reveals a loose net of imprecision, indeterminacy and subjectivity, which can be used as the basis of negotiations between powerful economic actors and the State*” (n.p.). Olssen (2008, p. 214) also argues that “for Foucault, democracy is the alternative to war, for democracy, is nothing but the tactics adopted to resolve conflict, ensure more or less peaceful transitions of power, and to permit each individual their legitimate arena or space, whereby rights—both passive and active—can be exercised and maintained. In this sense, by invoking the normative in Foucault, we can see that democracy is the containment and management of war. Democracy is politics, and “*politics*” as Foucault (2003) says, inverting Clausewitz’s famous aphorism, “*is the continuation of war by other means*” (p. 15)” (214f). Democracy, therefore, is a conflict-resolution strategy adopted to negotiate power relations. Though an alternative to war, it is still a power struggle, but a civil form where one exerts power rather silently. Democracy reduces the costs of maintaining and exercising power, so it is much easier to get people to buy into it. Corporations, therefore, welcome (and co-opt) democratic procedures.

Given the tendency in democracy to secure vested interests, especially in (post)capitalist, neoliberal systems (see also Przeworski and Wallerstein, 1982; Christiano, 2010; Parvin, 2018 for the uneasy relationship between democracy and socio-economic status), and how large corporations capitalize on democratic leadership, it is not unexpected to find instances of democratic leadership manipulated to secure corporate and organizational interests. In other words, corporations might use democracy and its structure to secure their vested interests, in a way that reduces democracy to a mere aureate façade for hiding classical power mechanisms.

Silent dismissal, therefore, might be truly consistent with an understanding of democracy as a regulating strategy aiming to secure economic privileges or a system of conflict resolution in power structures. Instead of equitably sharing power and encouraging equal participation, democracy here enlists accomplices in stigmatization and exclusion.

## Conclusion: Driven by External Acknowledgment

An explorative study, this paper deepens our understanding of (constructive) dismissals in new work environments and sheds new light on the subtle mechanisms of dismissing (visible) managers to circumvent litigation while upholding seemingly democratic principles and organizational values. Taking a critical stance toward the first-person narrative of managers, we conveyed that silent dismissal is not an isolated and occasional event; on the contrary, it is woven into organizational structures as a regulating strategy. Some aspects are similar to constructive dismissal like the personal perception of an unbearable work environment. Nevertheless, understandings of constructive dismissal so far imply (partly visible and legally traceable) harassment, threat, and hatred in the work environment, while silent dismissal seems effective because it is covered in seemingly soft or even favorable conditions. It indicates how *symbolic acknowledgment*, *social recognition*, and a sense of *belonging* are crucial circumstances for subjects, and how withholding them can turn into a compelling reason to resign. This can function as a subtle mechanism for dismissing subjects as it is virtually impossible to bring legal action against not being acknowledged.

Explicating this mechanism and contextualizing reveal that seemingly positive changes (such as a raise in salary or promotion) can be part of the dismissal process. Locating these dynamics within power structures makes it possible to make better sense of the reactions of all subjects involved and affected by the process of silent dismissal. Our findings enhance the state of knowledge by exposing the ethical and legal challenges of dismissals and foregrounding the vulnerability of employees when seeking justice. These novel insights call for juridical considerations and reflection on the justice of decision-making processes. This study further exposes new work as yet another possible facade for long-established power mechanisms, where seemingly progressive and equitable values are co-opted to serve economic and corporate interests. We have also suggested a potential avenue for bringing about change when we conveyed that the efficacy of silent dismissal depends on collective compliance. In other words, if individuals withdraw their complicity and take a critical and ethical stance toward the dynamics of power in organizations, there is a chance that noble values are less frequently assimilated to secure corporate interests over those of individuals.

Our findings, however, are not safely generalizable. This research used a case study method, and although two candidates were followed for 6 months, it might not be sufficient to observe the process only through the perspective of upper managerial positions. Therefore, the attitudes and reactions of other broader roles in organizations toward silent dismissal remain to be further researched to provide a more complete perspective on silent dismissals. It also remains to be studied how far this type of dismissal applies to other organizations (in terms of size, location, organizational structure, and legal context) and to stakeholders beyond managers (e.g., employees). On the last note, this contribution might inspire therapeutic processes, as the participants reported back that the methods of introspection, trained observation, and continuous guided reflections were on the one hand “somehow healing” (B 3; 821) and on the other hand, lead to envisioning of change in a critical psychological tradition (in terms of Frankfurter School) first steps or changed behavior and broadened scope: “I feel this process of intensive reflection brought me closer to my values and stance, maybe next time I speak up” (A 3; 821).

## Data Availability Statement

The original contributions presented in the study are included in the article/Supplementary Material, further inquiries can be directed to the corresponding author.

## Ethics Statement

Ethical review and approval was not required for the study on human participants in accordance with the local legislation and institutional requirements. The patients/participants provided their written informed consent to participate in this study.

## Author Contributions

JD collected data and took the lead for the analysis and method. MZ contributed in great manner to the theoretical frame and interpretation of the meaning of the findings. All authors contributed to the article and approved the submitted version.

## Funding

We acknowledge financial support by Schleswig-Holstein State within the funding program Open Access-Publication Fund. This work was also supported by the Alexander von Humboldt Stiftung/Foundation.

## Conflict of Interest

The authors declare that the research was conducted in the absence of any commercial or financial relationships that could be construed as a potential conflict of interest.

## Publisher’s Note

All claims expressed in this article are solely those of the authors and do not necessarily represent those of their affiliated organizations, or those of the publisher, the editors and the reviewers. Any product that may be evaluated in this article, or claim that may be made by its manufacturer, is not guaranteed or endorsed by the publisher.

## Footnotes

^1^As seen with phenomena such as green- and pinkwashing (Tomlinson and Schwabenland, 2010; De Luca et al., 2017; Vassilopoulou, 2017).

^2^Authors’ translation in collaboration with Scott Simpson as English editor, original data in German.

^3^According to Holzkamp, subject is to be understood as a general subject, not as one individual (Schraube and Osterkamp, 2013).

^4^“f” means from the line named and following down.

^5^The job title is of symbolic importance in the context of Germany.
