# Supplementary material for: Holding Up a Democratic Facade: How ‘New Work Organizations’ Avoid Resistance and Litigation When Dismissing Their Managers
Source: Front Psychol. 2022 May 6;13:789404. doi: 10.3389/fpsyg.2022.789404 (PMC9120358; doi:10.3389/fpsyg.2022.789404)
Supplement: Supplementary file 1 [file Table_1.docx]

Appendix/ Supplementary material

Table 1: *Comprehensive code tree*

| Category | Subcategory  1st order | Subcategory 2nd order | precision/ differentiation |
| --- | --- | --- | --- |
| 1 formal indicators and process of dismissal | 1.1 Visible process (explicit) | 1.1.1 progressive | - Physical relocation (change of office) - New position (same level or upwards) - Sharing positions/introducing new colleagues with the same tasks |
|  |  | 1.1.2 sudden | - Vanishing of the person - Restructuring: Disappearance of position - Repatriation: the disappearance of position |
|  | 1.2 invisible process/ informally (implicit) |  | - Exclusion from social events - Exclusion from communication - Changed atmosphere |
|  | 1.3 Target |  | - Executive management - Higher management - Middle management - Lower management |
| 2 Circumstances | 2.1 remaining key points |  | - Salary - Devices/technical equipment - Physical presence required |
|  | 2.2 removed key points |  | - Responsibility - Objective/task - Staff - Budget - Access to information - Desirable office space - Visibility of an audience - Procuration - Attention - Voice |
|  | 2.3 added key points |  | - Time (task-free presentism) - Salary (sometimes a raise) |
| 3 Communication of the dismissal | 3.1 silence/ none-  communicated | 3.1.1 official | - No communication |
|  |  | 3.1.2 unofficial | - Privately between remaining employees on a short and superfluous level |
|  | 3.2 general phrased yet explicit communication |  | - Informative internal email (general phrases according to situation) - External communication with press releases (highest ranks) |
| 4 Power mechanisms | 4.1 Perception/ mechanisms Level of the dismissed |  | - Exclusion/isolation - Exclusion from communication - Experience of being meaningless/ exchangeable - Uncertainty about the reason/ perceived randomness - Stigmatization - Fear of losing existential base and career |
|  | 4.2 Perception/ mechanisms: Level of the survivors |  | - Uncertain of being in favor - Lack of communication and information - Uncertainty about cause/ perceived randomness - Anxiety to lose existential ground |
| 5 Initiation & direction | 5.1 Intentional/ coming from a concrete source |  | - Executive management - Board - Management |
|  | 5.2 Sealed/diffuse direction/ uncertain |  | - Unknown - Secretary - Everybody |
| 6 Reactive behavior | 6.1 Dismissed subjects |  | - silent/ complying |
|  |  |  | - restrictive agency (one exception) |
|  | 6.2 Remaining employees | accepting/ neutral | - silent/ complying |
|  |  | accepting/ annoyed | - silent/ complying/ yet disagreeing with the practice |
